# Efficacy and Safety of Trametinib in Neurofibromatosis Type 1-Associated Plexiform Neurofibroma and Low-Grade Glioma: A Systematic Review and Meta-Analysis

**DOI:** 10.3390/ph15080956

**Published:** 2022-07-31

**Authors:** Dun Wang, Lingling Ge, Zizhen Guo, Yuehua Li, Beiyao Zhu, Wei Wang, Chengjiang Wei, Qingfeng Li, Zhichao Wang

**Affiliations:** 1 Department of Plastic and Reconstructive Surgery, Shanghai Ninth People’s Hospital, Shanghai Jiao Tong University School of Medicine, Shanghai 200011, China; wdiamond126@gmail.com (D.W.); gelinglingscu@foxmail.com (L.G.); gzzhen2016-@sjtu.edu.cn (Z.G.); lyh_discipline@163.com (Y.L.); beiyao.zhu@sjtu.edu.cn (B.Z.); dr_wangwei60@163.com (W.W.); licht001@outlook.com (C.W.); 2 Department of Burn and Plastic Surgery, West China Hospital of Sichuan University, No 37 Wainan Guoxue Road, Chengdu 610041, China

**Keywords:** neurofibromatosis type 1, plexiform neurofibroma, low-grade Glioma, trametinib, systematic review, meta-analysis

## Abstract

Trametinib has been used in neurofibromatosis type 1 (NF1) patients, especially those with unresectable nerve tumors, but no systematic review based on the latest studies has been published. We conducted this meta-analysis to evaluate the effectiveness and safety of trametinib in treating NF1-related nerve tumors. Original articles reporting the efficacy and safety of trametinib in NF1 patents were identified in PubMed, EMBASE, and Web of Science up to 1 June 2022. Using R software and the ‘meta’ package, the objective response rates (ORRs) and disease control rates (DCRs) were calculated to evaluate the efficacy, and the pooled proportion of adverse events (AEs) was calculated. The Grading of Recommendations, Assessment, Development and Evaluation system was used to assess the quality of evidence. Eight studies involving 92 patients were included, which had a very low to moderate quality of evidence. The pooled ORR was 45.3% (95% CI: 28.9–62.1%, I^2^ = 0%), and the DCR was 99.8% (95% CI: 95.5–100%, I^2^ = 0%). The most common AEs was paronychia, with a pooled rate of 60.7% (95% CI: 48.8–72.7%, I^2^ = 0%). Our results indicate the satisfactory ability to stabilize tumor progression but a more limited ability to shrink tumors of trametinib in NF1-related nerve tumors. The safety profile of trametinib is satisfactory.

## 1. Introduction

Neurofibromatosis type 1 (NF1) is a relatively common autosomal dominant genetic disorder, affecting about 1 in 3000 newborns, and is caused by mutations of the *NF1* gene, located on chromosome 17q11.2 [1,2,3]. NF1 is a tumor predisposition disease and is characterized by the growth of tumors on nerves throughout the body of an affected individual, potentially affecting the development of the brain, cardiovascular system, bones, skin and etc.

Plexiform neurofibromas (pNFs) are benign peripheral nerve sheath tumors that spread along the nerve in multiple fascicles that affect 25% to 50% of NF1 patients [4,5]. The compression of pNFs could lead to severe clinical complications, including pain, motor dysfunction, neurological impairment, and multiple organ damage [6]. More importantly, pNFs have malignant transformation potential to become malignant peripheral nerve sheath tumors (MPNSTs), which are the leading cause of NF1-related mortality [6,7,8,9]. The mainstay treatment for pNFs is surgical resection. However, due to the significant vascularization of the tumor and the tissue fragility, the risk of life-threatening intra- or postoperative bleeding has long been a tricky complication for surgeons [10,11,12]. In addition, especially for diffuse pNFs, complete surgical resection is often challenging due to the large extensive involvement and the encroachment and invasion of adjacent tissue [13]. According to surgical experience, the feasibility is also limited to a postoperative recurrence rate ranging from 1.3% to 54% [14,15,16].

Low-grade gliomas (LGGs), such as the most common central nervous system (CNS) tumors, affect nearly 20% of children with NF1 [17]. Among NF1 patients, LGGs typically occur within the optic pathway and can lead to progressive visual symptoms or other neurologic impairments [18]. The mainstay therapy for LGGs is also surgical excision, which is curative when total resection is possible. However, the execution of resection surgery is mainly restricted by the hardly accessible anatomical location, including the optic pathway, thalamus, and brainstem. Thus, for patients with unresectable LGGs or classified as high risk, adjuvant therapy with radiation or chemotherapy is still needed to control the recurrence or progression. However, radiation exposure is an independent risk factor for MPNST, which is a typical malignancy in NF1 patients, so it is not applicable to these patients [19]. The most used chemotherapy regimens are temozolomide (TMZ) alone or a combination of procarbazine/lomustine/vincristine (PVC) [20,21,22,23,24]. Although effective, both regimens are associated with grade three and four toxicities such as secondary malignancy and infertility. In addition, the long-term efficacy is still controversial, with the 5-year progression rate reaching 30% [25,26].

The *NF1* gene encodes the tumor suppressor protein neurofibromin. It inhibits RAS activation and the downstream RAS-mitogen-activated protein kinase (MAPK) pathway, which plays a vital role in cell differentiation and proliferation [27]. In NF1 patients, the dysfunction of neurofibromin would lead to overactivation of the RAS-MAPK pathway [28]. Thus, the mitogen-activated protein kinase (MEK) inhibitors potentially offer a novel option for unresectable pNFs and LGGs. Trametinib (GSK 1120212) is a MEK1/2 inhibitor that could limit the abnormal activation of the RAS pathway by inactivating the MAPK kinase (MEK), and it is historically used to treat BRAF-mutant melanoma and non-small-cell lung cancer [29,30]. There is one ongoing phase II trial investigating the benefit of trametinib for treating LGGs and pNFs in NF1 individuals [31], and other reports on trametinib for treating NF1-related nervous system tumors have also been published in recent years [25,32,33,34,35,36,37,38,39,40]. However, due to the mixed inclusion criteria, and the limited sample size, the strength of the evidence remains open to question. Thus, we conducted this comprehensive and systematic meta-analysis of published data on the efficiency and safety of the trametinib for treatment of NF1-related nervous tumors.

## 2. Results

### 2.1. Study Characteristics and Quality Assessment

**Figure 1:**
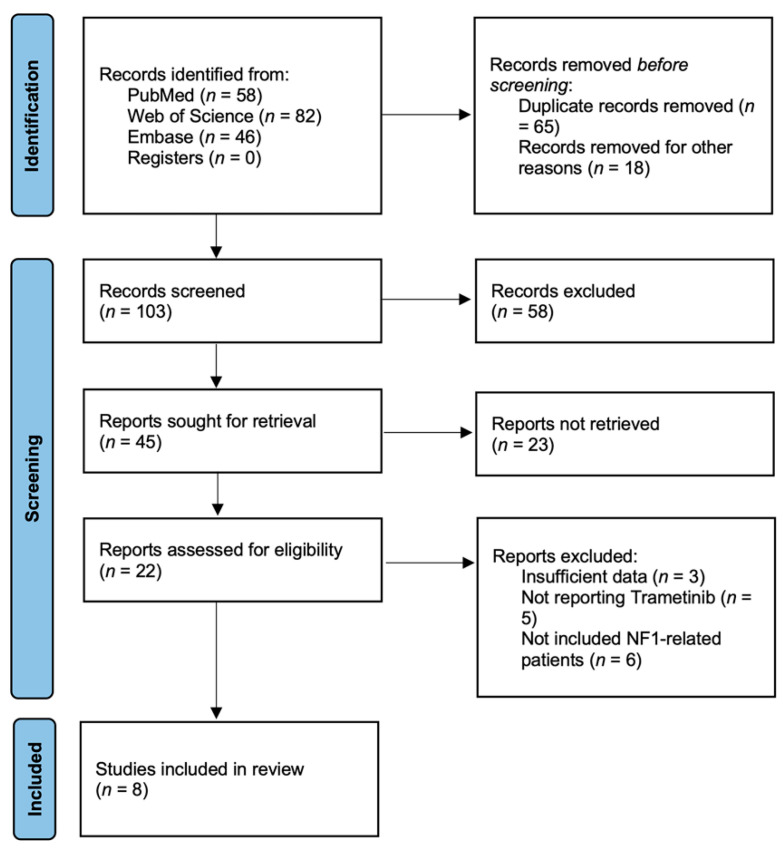
Flow chart showing the process of identification of selected studies.

The search strategy initially retrieved 163 potentially relevant clinical studies. A total of eight studies published between 2018 and 2021 were included [25,32,33,34,35,36,37,38]. None of the studies were randomized controlled. The flow chart of the reference selection is shown in Figure 1. The general characteristics and quality assessments of studies included in the meta-analysis are presented in Table 1. Two studies were phase I clinical trials [35,37], and six studies were retrospective case series [25,32,33,34,36,38]. The eight studies included 127 patients in total. Ninety-four patients were included in our meta-analysis since we only focused on the NF1-related patients. All the studies used MRI to assess treatment response by imaging, and the criteria used to assess response based on imaging were presented in Table 2. Two studies reported patients with LGGs [25,33], five studies reported patients with pNFs [34,35,36,37,38], and one study included both LGG and pNF lesions [32]. Three studies were carried out in the USA [25,36,38], one in Canada [32], one in Germany [33], one in Israel [34], one in Australia [35], and one in France [37].

The quality of the eight studies included was initially assigned as ‘low’ given their observational nature (two of them were non-randomized clinical trials [35,37], and the other six were all retrospective studies [25,32,33,34,36,38]). One study was then downgraded to “very low” for not clearly defining the radiological response, which could be biased [38]. Three studies were upgraded to “moderate” due to the effect size with a cut-off value of effect size (ORR) = 0.5 [32,33,36]. The result of the quality assessment is presented in Table 3.

### 2.2. Efficacy

**Figure 2:**
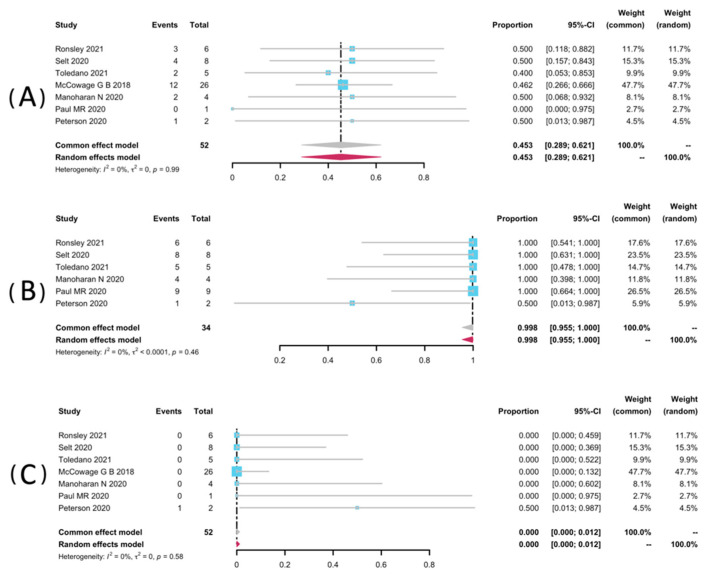
Forest plots for the objective response rate (**A**), the disease control rate (**B**), and the progression rate (**C**) in patients receiving trametinib.

**Figure 3:**
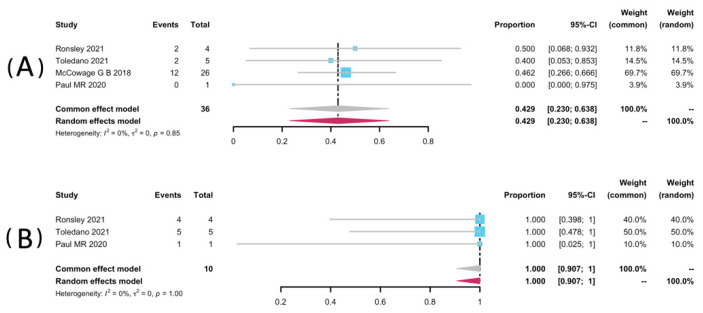
Forest plots for the objective response rate (**A**), and the disease control rate (**B**) in pNFs patients receiving trametinib.

**Figure 4:**
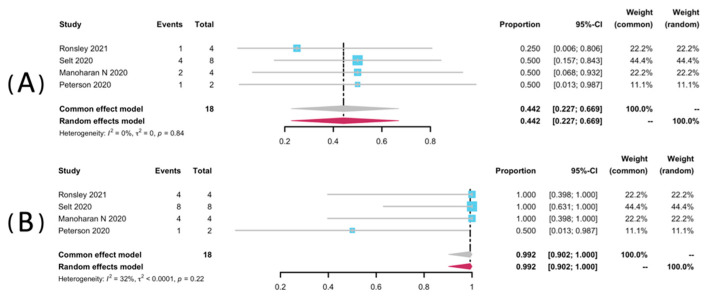
Forest plots for the objective response rate (**A**), and the disease control rate (**B**) in LGGs patients receiving trametinib.

Volumetric analyses of 52 patients in seven studies [25,32,33,34,35,36,38] were extracted to pool the ORR, and 34 patients from six studies were analyzed for DCR [25,32,33,34,36,38]. The result of pooled ORR is 45.3% (95% CI: 28.9–62.1%) with no significant heterogeneity (*p* = 0.99, I^2^ = 0%). The pooled DCR was 99.8% without significant heterogeneity (*p* = 0.46, I^2^ = 0%). Among the included studies, disease progression was observed in only one study (1/2, 50%), yielding a pooled rate of zero (95% CI: 0.0–0.01%, I^2^ = 0%) [25]. The forest plots of pooled ORR, DCR, and progression rate were presented in Figure 2. Subgroup analyses were undertaken by including only patients with pNFs (Figure 3) and LGGs (Figure 4). The overall result remains steady with the ORR of 42.9% (95% CI: 23.0–63.8%, I^2^ = 0%) and 44.2% (95% CI: 22.7–66.9%, I^2^ = 0%), and the DCR of 100% (95% CI: 90.7–100.0%, I^2^ = 0%) and 99.2% (95% CI: 90.2–100.0%, I^2^ = 0%), respectively.

### 2.3. Safety

**Figure 5:**
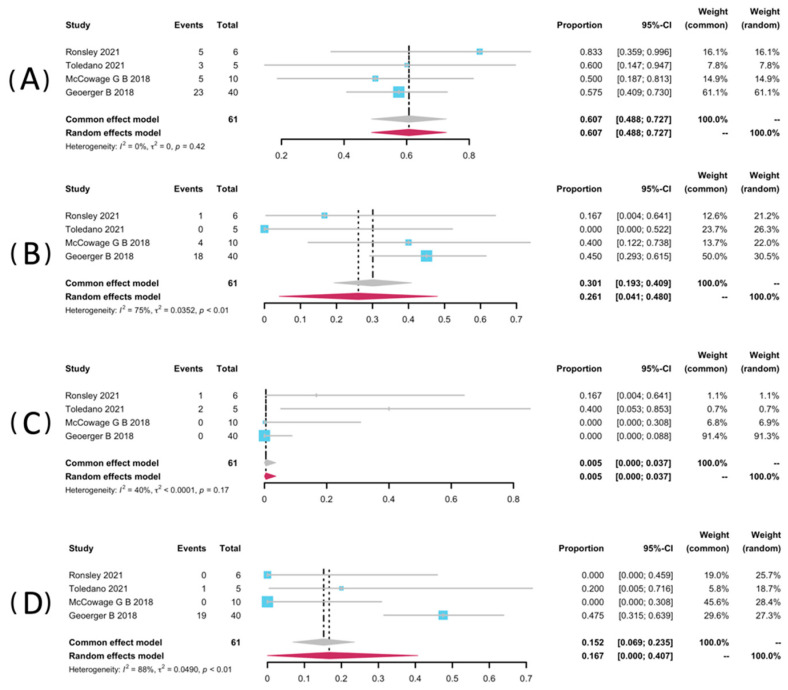
Forest plots for the adverse events paronychia (**A**), rash (**B**), mouth ulcer (**C**), and diarrhea (**D**) in patients receiving trametinib.

Restricted to NF1-related patients, data of AEs of any grade were pooled from four studies [32,34,35,37]. Among these three studies, they used Common Terminology Criteria or Adverse Events (CTCAE version 4.0) [34,35,37], and another one used version 5.0. Data from five studies were not pooled for the safety analyses because they did not report NF1-related patients as a separate group [25,33,36,38]. AEs analyzed include rash, paronychia, and diarrhea. The most frequent AE was any grade of paronychia, with a pooled rate of 60.7% (95% CI: 48.8–72.7%, I^2^ = 0%). It is worth mentioning that Ronsley et al. and Toledano et al. each reported a case requiring revision or discontinuation of a treatment regimen resulting from severe paronychia [32,34]. Selt and co-workers reported an occurrence of 7/18 (38.9%) of paronychia during the trametinib treatment among LGG patients with-or-without NF1 [33]. The prevalence of all grades of rash was 26.1% (95% CI: 19.3–40.9%, I^2^ = 75%). It was also close to that in Selt’s study, which was 27.8% (5/18) [33]. In addition, diarrhoea presented a pooled rate of 16.7% (95% CI: 0.00–40.7%, I^2^ = 88%). In addition, the pooled rate of mouth ulcer was 0.5% (95% CI: 0.0–3.7%, I^2^ = 40%). The pooled forest posts of AEs are presented in Figure 5.

## 3. Discussion

The results yielded in this systematic review and meta-analysis showed that trametinib significantly controlled the tumor progression of NF1-related pNF and LGG, with a pooled DCR of 99.8%. Apart from the efficacy on lesion stability, an acceptable range of side effects and manageable safety level both supported trametinib as a new choice in the treatment of NF1 individuals with nervous system neoplasms.

However, when compared to another MEK inhibitor, selumetinib (AZD6244, ARRY-142886), which was recently approved to treat children’s NF-1-related symptomatic, inoperable pNFs [43], the capacity of tumor amelioration is barely satisfactory with the ORR of 44.2% versus 73.8% [44]. Nonetheless, the advantages of trametinib over selumetinib in terms of the dosage form are noteworthy. Selumetinib is currently available only in a non-dissolvable form and therefore has a limitation in the treatment of very young children for whom the intact tablets are hard to swallow. Considering the susceptibility of NF1-related nervous system tumors at an early age, trametinib could suit a wider age range of the population since it is available as a suspended powder [34]. Indeed, the minimum age for the trametinib treatment can be less than 6 months old, in comparison to the minimum age of 3-year-old in their selumetinib counterpart [25,44].

As for NF1-related LGGs, our study demonstrated good capability in tumor suppression of trametinib, with the DCR reaching 99.2%, and the objective response rate is yielded as 44.2%. When compared to chemotherapy, the classic non-surgical treatment strategy of LGGs, the prominent advantage of trametinib is its tolerability. Procarbazine is associated with primary hematologic toxicity such as nausea, vomiting, pancytopenia, and even a 2–15% risk of secondary malignancy, and Vincristine is associated with a primary concern of neurotoxicity [45,46,47]. In a retrospective series of 57 LGG patients receiving PCV, 28% of the patients presented thrombocytopenia, 7% of the patients presented grade 3 or higher anemia, and neurotoxicity was observed in 60% of the patients [48]. In contrast, the adverse events observed in the current study are relatively mild. Consistent with previous reports of both trametinib and selumetinib, skin and nail toxicities were the most observed but were always reversible by stopping the medication or anti-inflammatory management [49,50,51]. Recently, the use of MEK inhibitors in the management of LGG patients is being tested in ongoing clinical trials, including selumetinib (NCT01089101) and trametinib (NCT02124772). Further findings with long-term survival data are still needed to confirm the value of MEK inhibitors in the treatment of NF1-related LGGs.

In this systematic review and meta-analysis, we showed the considerable efficacy and safety of trametinib treatment of inoperative NF1-LGGs and refractory NF1-pNFs, with a wider application range from newborn children to young adults. The clinical benefits were reflected in the minimization of tumor size, clinical symptoms remission, and function improvement. The better clinical outcomes were found in the youngest patients by Ronsley et al. [32]. In addition, Toledano et al. reported a result without obvious improvement in terms of visual symptoms, claiming that the treatment with MEK inhibitors should be started earlier than the irreversible visual decline [34]. Thus, further studies on a larger scale are needed to investigate the relationship between the time of treatment initiation and treatment outcome. In conclusion, the individual studies included all showed positive effects of tumor volume shrinkage in the majority of the enrolled patients. Although it has been proven to be an effective medical targeted therapy, the optimal duration of treatment remains unknown and requires longer follow-up data.

Other MEK inhibitors have also emerged in the field of NF1-patient therapy in recent years. A phase II trial using Mirdametinib (PD0325901) in treating inoperable pNFs presented its potential capacity in the adult population (≥16 years old) for the first time, with an ORR of 42%, as well as preliminary evidence of a reduction in pain. Throughout the full cycle of treatment, a time-dependent trend in tumor size shrinkage was observed [52]. In 2021, Pérez et al. retrospectively studied the efficacy and tolerability of trametinib and dabrafenib in pediatric non-NF1 LGGs [53]. As the results showed, dabrafenib seems to be more effective in this population than trametinib (ORR: 41.7% versus 0%; DCR: 100% versus 78.6%). However, the determination of specific medical choice and therapeutic duration for NF1-related and NF1-unrelated individuals of different age groups still calls for further investigations with a larger sample size.

In the trials using MEK inhibitors to treat NF1 patients, the most reported side effects include digestive reaction, skin toxicities, and elevation in creatinine kinase levels [54]. In the studies included in this meta-analysis, treatment with trametinib could be complicated by grade 1 and 2 AEs, of which the most frequently observed one was any grade of paronychia with a pooled rate of 60.7%, followed by the appearance of all grades of rash with a pooled rate of 26.1%, both as typical manifestations of dermatologic toxicity, while no long-term or irreversible skin or nail toxic effects were observed in all the trails. Overall, trametinib demonstrated a manageable safety profile in patients with unresectable NF1-LGGs or life-threatening NF1-pNFs.

This meta-analysis has some limitations, and therefore, the results should be interpreted with caution. First, since trametinib is not routinely used in the treatment of NF1 patients, we are only able to retrieve a small number of studies, which would lead to a lack of statistical power. Secondly, the sample sizes of the included studies were too small to be divided into different age groups. Given that pNFs tend to grow faster in younger children [55,56], further studies with patient-level data could enable more accurate analysis results. Third, the current analysis failed to pool data on some clinical results such as pain-relieving and improvement of neurological symptoms due to the different measurements and lack of initial data. Fourth, only Selt et al. and Toledano et al. reported the method used for volumetric analysis [33,34]. The image acquisition and volumetric analysis method should be standardized in order to assess tumor volume changes more sensitively and reliably [57].

In conclusion, the current systematic review and meta-analysis demonstrated the efficacy and safety of trametinib for pediatric patients with NF1-related, symptomatic, inoperable pNFs and LGGs. Further large-scale, randomized controlled trials are needed to confirm our current result.

## 4. Materials and Methods

We conducted this systemic review and meta-analysis according to the latest Preferred Reporting Items for Systematic Reviews Meta-Analyses guidelines and PRISMA statement 2020 [58]. The review and meta-analysis were registered (registration number: CRD42022338481) with the International Prospective Register of Systematic Reviews (PROSPERO).

### 4.1. Systematic Literature Search

A systematic search of three main databases, including PubMed, Embase, and Web of Science, was carried out to retrieve the articles published up to 1 June 2022. The search strategies were presented in Figure S1. Articles were included only if they were human studies published in the English language with full-text descriptions. Reference lists from retrieved articles were also examined to identify relevant studies.

### 4.2. Inclusion Criteria and Exclusion Criteria

Two reviewers (D.W. and L.L.G.) screened and identified the search findings for potentially eligible studies. The inclusion criteria were as follows:(1) clear documentation of the patients diagnosed with NF1-related pNF or LGG; (2) original articles reporting data on the clinical efficacy or safety of trametinib; (3) studies performing imaging examinations (magnetic resonance imaging, MRI or computed tomography, CT) to evaluate the treatment response; (4) studies reported in the English language; (5) when multiple studies were published by the same institution or authors, either the higher-quality study or the most recent publication was included.

The following studies were excluded:(1) abstracts, letters, expert opinions, and reviews; (2) studies with no reported outcomes of interest; (3) studies with insufficient data to extract; (4) studies reporting MEK inhibitors other than trametinib or combined use of trametinib with other therapies in the treatment of NF1 patients.

Two independent reviewers (D.W. and L.L.G.) determined the final inclusion of articles; a third author adjudicated when this failed.

### 4.3. Data Extraction and Quality Assessment

One independent reviewer (D.W.) extracted the data using standardized forms, and another reviewer (L.L.G.) checked the collected data. Any disagreements were resolved by consensus. The recorded data from the selected study included: (1) study characteristics (author, year of publication, institution, study design); (2) patient characteristics (patient number, age, gender, inclusion criteria, tumor location, treatment, target tumor location, percentage of progressive disease at enrollment); (3) imaging characteristic (modality, the plane of acquisition, sequences, the time interval of imaging, criteria of responses); (4) imaging response (complete response, partial response, minor response, stable disease, disease progression); (5) type and several adverse events (AEs).

The quality of studies was assessed using the Grading of Recommendations, Assessment, Development and Evaluation (GRADE) system [59]. In this system, the quality of the studies was initially evaluated based on the study design. After which, the quality may upgrade based on moderate/large effects, dose responses, plausible confounding factors, and may downgrade based on risks of bias, inconsistencies, indirectness, imprecision, and publication bias. The final quality of the studies would be graded as either ‘very low’, ‘low’, ‘moderate’, or ‘high’.

### 4.4. Statistical Analysis

The efficacy was evaluated using objective response rate (ORR; the proportion of the patients presenting complete response, partial response, and minor response to the trametinib treatment) and disease control rate (DCR; the proportion of patients with complete response, partial response, minor response, and stable disease). For safety analyses, reports of AEs of any grade were collected and evaluated using the Common Terminology Criteria or Adverse Events (CTCAE) [60].

R version 4.2.0 and the R package ‘meta’ were used for performing the meta-analysis and generating the forest plots (R Foundation for Statistical Computing) [61]. Odds ratio (OR) was used with a corresponding 95% confidence interval (CI) to analyze the variables that we included. The pooled effects were calculated using both common-or random-effects models. Heterogeneity was evaluated by I^2^ with *p* < 0.1 taken as significant [62]. Sensitivity analyses were also performed by excluding individual studies from the data set to analyze their relative effects on the overall pooled estimates. Publication bias was not conducted because fewer than 10 studies were included [63]. Due to the nature of our study, no ethical approval and patient consent were required.

## Figures and Tables

**Table 1:** Study and patient characteristics.

**Trametinib Dosage**	NR	0.025 mg/kg (*n* = 3), 0.032 mg/kg (*n* = 2), 0.016 mg/kg (*n* = 1) Daily	0.03 mg/kg Daily	0.032 mg/kg Daily	0.025 mg/kg (*n* = 21), 0.032 mg/kg (*n* = 1), 0.040 mg/kg (*n* = 4) Daily	0.025 mg/kg Daily	0.025 mg/kg 0.04 mg/kg Daily	0.025 mg/kg Daily
**Tumor**	LGG	pNF and LGG	LGG	pNF	pNF	pNF	pNF	pNF
**Age, year (Range)**	Median 3 (0.5–6.8)	Median 9 (1–14)	Median 2.1 (0.5–9.9)	Median 2.3 (0.5–3.2)	Median 5.5 (1–16)	Median 14.7 (7.3–25.9)	Median 8 (0–18)	Median 10.8 (5.2–17.1)
**Included Patients number**	2	6	8	5	26	4	40	1
**Patient Number (Male/Female)**	8 (5/3)	6 (3/3)	18 (8/10)	5 (1/4)	26	10 (6/5)	40	14 (9/5)
**Enrollment period**	2014–2019	2017.12–2020.5	2015–2019	2016.1–2018.8	2014.4–2021.6	2016–2018	2014.4–2021.6	2015.1–2019.9
**Country**	USA	Canada	Germany	Israel	Australia	USA	France	USA
**Institution**	Multicenter	University of British Columbia	Multicenter	Multicenter	Multicenter	Multicenter	Multicenter	Multicenter
**Study**	Peterson et al. 2020 [25]	Ronsley et al. 2021 [32]	Selt et al. 2020 [33]	Toledano et al. 2021 [34]	McCowage et al. 2018 [35]	Manoharan et al. 2020 [36]	Geoerger et al. 2018 [37]	Paul et al. 2020 [38]

NR: Not reported.

**Table 2:** Response criteria in included studies.

Study	Criteria
Ronsley et al., 2021 [32]	LGG: Partial response: decrease ≥50% Minor response: decrease 25–49% Progression: increase ≥25% pNF: Partial response: decrease ≥20% Progression: increase ≥20%
Selt et al., 2020 [33]	Complete response: no evidence of residual or recurrent tumor or dissemination Partial response: decrease ≥50% Minor response: decrease 25–50% without new lesions Stable disease: change in volume between +25–−25% without new lesions Progression: increase ≥25% or appearance of new lesions
Toledano et al., 2021 [34]	NA
McCowage et al., 2018 [35]	Dombi criteria [41]
Manoharan et al., 2020 [36]	RANO criteria [42] Minor response: decrease 25–50%
Peterson et al., 2020 [25]	Radiological response, radiologically stable, and progression without clear definition
Geoerger et al., 2018 [37]	Dombi criteria [41]
Paul et al., 2020 [38]	Complete response, partial response, stable disease, progressive disease without clear definition.

LGG: low-grade glioma; pNF: plexiform neurofibroma.

**Table 3:** GRADE table for this meta-analysis.

Quality of Evidence	Low	Moderate	Moderate	Low	Low	Moderate	Low	Very Low
Dose effect	NA	NA	NA	NA	NA	NA	NA	NA
Effect size	Moderate	Moderate	Moderate	Small	Small	Moderate	NA	Small
Publication bias	√	√	√	√	√	√	√	√
Imprecision	√	√	√	√	√	√	√	√
Indirectness	√	√	√	√	√	√	√	√
Inconsistency	√	√	√	√	√	√	√	√
Risk of bias	×	√	√	√	√	√	√	×
Study design	Cases	Retrospective	Retrospective	Retrospective	Non-randomized clinical trial	Non-randomized clinical trial	Non-randomized clinical trial	Retrospective
Study	Peterson et al. 2020 [25]	Ronsley et al. 2021 [32]	Selt et al. 2020 [33]	Toledano et al. 2021 [34]	McCowage et al. 2018 [35]	Manoharan et al. 2020 [36]	Geoerger et al. 2018 [37]	Paul et al. 2020 [38]

√ indicates no serious limitations; ×, serious limitations; effect size—objective response rate (ORR) ≥ 0.5 for moderate effect or ORR ≥ 0.8 for large effect; NA, not applicable.

## Data Availability

Not applicable.

## Supplementary Materials

The following supporting information can be downloaded at: https://www.mdpi.com/article/10.3390/ph15080956/s1, Figure S1: Searching Strategy. (**A**) PubMed search strategy; (**B**)Web of Science search strategy; (**C**) Embase search strategy.
