# Supplementary material for: Efficacy and Safety of Trametinib in Neurofibromatosis Type 1-Associated Plexiform Neurofibroma and Low-Grade Glioma: A Systematic Review and Meta-Analysis
Source: Pharmaceuticals (Basel). 2022 Jul 31;15(8):956. doi: 10.3390/ph15080956 (PMC9415905; doi:10.3390/ph15080956)
Supplement: Supplementary file 1 [file pharmaceuticals-15-00956-s001.zip › pharmaceuticals-1807246-supplementary.pdf]

(**A**) PubMed search strategy

#1 Trametinib

#2 Neurofibromatosis type 1 [Title/Abstract]

#3 NF1 [Title/Abstract]

#4 #2 OR #3

#5 plexiform neurofibroma [Title/Abstract]

#6 pNF [Title/Abstract]

#7 #5 OR #6

#8 low-grade glioma [Title/Abstract]

#9 LGG [Title/Abstract]

#10 #8 OR #9

#11 #7 OR #10

#12 #1 AND #4 AND #11

(**B**) Web of Science search strategy

#1 (TS=(NF1)) OR TS=(neurofibromatosis type 1)

#2 (TS=(pNF)) OR TS=(plexiform neurofibroma)

#3 (TS=(LGG)) OR TS=(low-grade glioma)

#4 TS=(Trametinib) AND #1 AND (#2 OR #3)

(**C**) Embase search strategy

#1 exp Trametinib/

#2 exp Neurofibromatosis type 1/

#3 exp NF1/

#4 #2 OR #3

#5 exp plexiform neurofibroma/

#6 exp pNF/

#7 #5 OR #6

#8 exp low-grade glioma/

#9 exp LGG/

#10 #8 OR #9

#11 #7 OR #10

#12 #1 AND #4 AND #11

Figure S1. Searching Strategy. **(A)** PubMed search strategy; **(B)**Web of Science search strategy; **(C)** Embase search strategy.
